# Association between chronic low back pain and regular exercise, sedentary behaviour and mental health before and during COVID-19 pandemic: insights from a large-scale cross-sectional study in Germany

**DOI:** 10.1186/s12891-022-05806-8

**Published:** 2022-09-15

**Authors:** M Hochheim, P Ramm, M Wunderlich, V Amelung

**Affiliations:** 1 Institute of Epidemiology, Medizinische Hochschule Hannover (MHH), Social Medicine and Health System Research, Carl-Neuberg-Straße 1, 30625 Hannover, Germany; 2 Generali Health Solutions GmbH (GHS), Hansaring 40 – 50, 50670 Cologne, Germany

**Keywords:** Low back pain, Humans, Graded chronic pain scale, COVID-19, Odds ratio, Exercise, Sedentary behaviour, Mental health, Insurance, Health, Germany

## Abstract

**Background:**

Nonspecific chronic low back pain (CLBP) is a complex symptom with numerous possible causes and influencing factors. Understanding how modifiable factors affect the course of CLBP is important for preventing progression. As the COVID-19 pandemic has changed the lifestyle of many people, this study paper assessed whether it also changed the influence of modifiable lifestyle factors (regular exercise and sedentary behaviour) and mental health factors (anxiety and depression) on CLBP pain intensity and disability by comparing the strength of these associations before and during the pandemic. We hypothesised that the importance of regular physical activity and good mental health for CLBP patients would increase during the pandemic.

**Methods:**

These questions were investigated in a cross-sectional study of insurance claims data and self-reported data from various questionnaires from 3,478 participants in a German CLBP health intervention (2014–2021) by calculating pre- and intra-pandemic odds ratios (OR) and 95% confidence intervals (CI) for each variable of interest and outcome. Potential confounders were also considered. Pandemic status was treated as an effect modifier. Based on the date of enrolment, participants were classified as “pre-pandemic” or “pandemic”.

**Results:**

Regularly exercising ≥ 4 h/week significantly reduced the odds of high disability for men (OR 0.49, 95% CI 0.31 – 0.79, *p* = 0.003) and women (OR 0.30, 95% CI 0.14 – 0.563, *p* = 0.002) and reduced the probability of severe pain in women (OR 0.37, 95% CI 0.21 – 0.65, *p* < 0.001). Each one-point increase in PHQ-4 score for anxiety and depression increased the OR of high pain intensity by 1.25 points (95% CI 1.18 – 1.34, *p* < 0.001). A clear impact of COVID-19 lockdowns was observed. In individuals who exercised ≥ 4 h/week the OR of high disability was 0.57 (95% CI 0.36 – 0.92, *p* = 0.021) in the pre-pandemic group compared to 0.29 (95% CI 0.12 – 0.56, *p* = 0.002) in the pandemic group. The probability of high disability increased from an OR of 1.42 (95% CI 1.33 – 1.52, *p* < 0.001) per marginal increase in the PHQ-4 scale before the pandemic, to an OR of 1.73 (95% CI 1.58 – 1.89, *p* < 0.001) during the pandemic.

**Conclusions:**

The magnitude of association of the factors that influenced high pain intensity and disability increased during the pandemic. On the one hand, the protective effect of regular exercising was greater in participants surveyed during lockdown. On the other hand, a higher risk through anxiety or depression during the lockdown was identified. An additional study with objective measures of sedentary behaviour and physical activity is needed to validate these results. More in-depth investigation of lockdown-induced associations between reduced daily physical activity, increased levels of anxiety and depression, and their effects on CLPB could also be worthwhile.

**Trial registration:**

This study used routinely collected data from a CLBP intervention that was previously evaluated and registered in the German Registry of Clinical Trials under DRKS00015463 (04/09/2018). The original ethics approval, informed consent and self-reported questionnaire have remained unchanged and are still valid.

**Supplementary Information:**

The online version contains supplementary material available at 10.1186/s12891-022-05806-8.

## Background

The outbreak of Coronavirus Disease 2019 (COVID-19) around the world led to social distancing, lockdowns and restrictions in more than 100 countries, resulting in drastic lifestyle changes [1]. Among other things, people were expected to work from home wherever possible, reorganise childcare, and reduce in-person social contacts. Public life was almost completely suspended in many places. Sports clubs, gyms, and pools were forced to temporarily shut down, limiting opportunities to exercise [2]. Pandemic restrictions increased social isolation and greatly impacted human behaviour within a very short period of time [3]. Physical activity levels decreased significantly while levels of sedentary behaviour, psychiatric morbidity and psychological distress increased [1, 4, 5]. Thus, the COVID-19 pandemic has caused changes in biopsychosocial and other factors at almost every level [6].

Nonspecific chronic lower back pain (CLBP) is defined as pain and discomfort localised below the costal margin and above the gluteal folds, with or without radiation to one or both legs, that persists for 12 weeks or more without a specific underlying cause [7]. CLBP is a complex symptom with many possible causes and influencing factors. Risk factors may be individual (e.g., age, sex, body mass index, lifestyle), social (e.g., education, work satisfaction), psychological (e.g., depression, fear of movement), and external (e.g., demanding manual work, carrying heavy loads, difficult working positions) [6, 8]. Modifiable risk factors can be divided into six categories: cognitive (e.g., self-belief, self-efficacy), coping (flexibility), emotional (e.g., anxiety behaviour, resilience), lifestyle (activity levels, sleep, body weight), physical (e.g., functional behaviour, body composition) and social (cultural, work and family environment) [9].

Whether the pandemic will ultimately benefit CLBP patients (more flexibility in work organisation, less travel stress, more opportunities to incorporate physical activity in the working day) or worsen their symptoms (lack of opportunity to perform structured supervised exercise, increased time stress due to new childcare tasks, lack of social support, poor ergonomic equipment at home) is not certain at this point in time. However, decreased use of treatment services as well as increased pain intensity and prevalence of CLBP have been reported during the pandemic [10–12]. A large statutory health insurance company in Germany recorded an eight percent increase in sick days due to back pain [13].

Knowing how the influencing factors for CLBP changed during the pandemic and whether these changes impacted the severity of CLBP is important for understanding the impact of COVID-19 on CLBP. Current research is highly focused on prognostic models to predict the first onset of low back pain or the development of chronicity [8, 14–22]. CLBP should be evaluated not only according to the presence and duration of symptoms, but also according to its effects on the affected individuals. The Graded Chronic Pain Scale (GCPS) by Von Korff et al., which contains subscales for pain intensity and pain-related physical disability, is a suitable instrument for this. Its scales can be used to divide CLBP disability into four grades, ranging from low (functional) to high (dysfunctional) [23]. Dysfunctional disability is associated with high levels of pain and suffering and is severely limiting [24]. Although dysfunctional disability is the least common form in CLBP, it causes the highest proportion of direct and indirect healthcare costs [25–27].

This research paper focuses on the effects of physical activity, sedentary behavior, and mental health on CLBP pain intensity and disability before and during the COVID-19 pandemic. By definition, physical activity is “*any bodily movement produced by skeletal muscles that requires energy expenditure”* [28], whereas sedentary behaviour is “*any waking behaviour characterised by an energy expenditure* ≤ *1.5 metabolic equivalents (METs), while in a sitting, reclining or lying posture”* [29]. These are diametrically opposed entities on the energy expenditure continuum [30]. Even after adjustment for physical activity, sedentary behaviour remains a major risk factor for chronic diseases such as type 2 diabetes, cardiovascular diseases and mental health disorders [31–35]. Public health agencies recommend 150 to 300 min of activity per week, yet many people still spend a large portion of the day sitting during leisure and occupational time (especially during a lockdown) [30].

As the COVID-19 pandemic has changed the lifestyle of many people, we suspect that this will affect the influence of modifiable lifestyle factors (regular exercise and sedentary behaviour) and mental health factors (anxiety and depression) on CLBP pain intensity and disability. We hypothesise that the importance of regular physical activity and good mental health for CLBP patients will increase in times of a massive transformation such as the pandemic.

Therefore, the objective of this study was to examine the strength of association of regular exercise, sedentary behaviour, and mental health factors with CLBP intensity and disability. The secondary objective was to compare these associations before and during the COVID-19 pandemic. An updated estimate of CLBP-related health care costs in Germany before and during the pandemic was generated as a by-product and secondary outcome of this research.

## Methods

### Study design

This was an observational cross-sectional study of retrospectively collected insurance claims data and self-reported data from various questionnaires from 3,478 participants in a German CLBP health intervention (2014–2021). The information was originally collected for different reasons (to settle claims and for eligibility assessment before participation in the health intervention). Claims data and self-reported questionnaire data were provided by Generali Deutschland Krankenversicherung AG (“Generali Deutschland”), a private health insurance company formerly known as “Central Krankenversicherung”. Pseudonymized data were used for the analysis. The results are presented according to the *Strengthening the Reporting of Observational Studies in Epidemiology (STROBE)* statement [36].

### Setting

Generali Deutschland is one of the largest private health insurance providers in Germany. In 2021, it had 298,210 fully insured members and 1,501,355 with supplementary partial insurance [37]. In the dual public–private health insurance system in Germany, privately insured usually belong to higher socio-economic classes and consist of self-employed persons, civil servants and employees with a salary above the compulsory insurance threshold, which is currently €64,350 [38–41]. Since 2014, Generali Deutschland has been operating a German-wide primary care intervention using a biopsychosocial approach that is offered proactively to clients suffering from CLBP. The intervention consists of a 12-month programme of spine muscle stabilising training, behavioural change telephone coaching, and patient education [42, 43]. Whereas training was conducted exclusively on-site at specific treatment centres before the pandemic, it had to be performed primarily with the Kaia mobile app [44–46] during pandemic lockdowns, when access to local training facilities was tightly restricted or closed [47].

### Participants

Study participants were all Generali members with comprehensive private health insurance, who were enrolled in the CLBP intervention between July 2014 and March 2021. They were located throughout Germany and had a minimum age of 18. There were two ways to participate in the intervention. The standard way was through an invitation from the insurance company. Invitations to participate in the health programme were based on the insured person's billing data. The invitees were selected so that CLBP was very likely. Before the pandemic, the classification tool by Freytag et al. was used to identify insured persons with CLBP [48]. Invited patients needed at least two billing events for ICD-10 M40-M54 disorders (dorsopathies) in combination with a) temporary work disability due to back pain or b) opioid prescription or c) mental health disorders within the last 12 months. The full list of criteria has been published elsewhere [42]. The alternative path was based on patient initiative (self-selected participants): In this case, the patient directly requested permission to participate in the CLBP health programme by contacting their insurance provider’s customer service by phone. The customer service assessed their duration of back pain by asking: “How long have you had back pain?” If the requester reported having low back pain for 12 weeks or more, they received an individual invitation to participate in the intervention.

The introduction of the Kaia app during the pandemic resulted in the lowering of entrance barriers. Invitees only needed two billing events for ICD-10 M40-M54 disorders within the last 12 months, and all individuals who requested to participate in the programme were granted access without further checking their duration of symptoms. Participants enrolled in the intervention before the first German lockdown were classified as “pre-pandemic” patients, and those enrolled on or after 22 March 2020 were classified as “pandemic” patients.

### Data sources and measurement

All participants in the long-term CLBP health programme underwent a standardised, digital self-assessment procedure consisting of multiple questionnaires before enrolment to ensure that they were eligible for the intervention. The self-assessment data from all health programme participants were used in combination with insurance provider claims data to answer the research question at hand. Demographic (sex, age, job status), health system utilisation (total health costs, back pain-specific costs, number of ICD-10 back pain-related [M*] and anxiety and depression-related [F*] diagnoses), and comorbidity type and severity data (Charlson's Comorbidity Index Score) were extracted from the health insurance system’s population and claims database from 2013 to 2021. Information required to calculate Graded Chronic Pain Scale (GCPS) scores [23, 49] and to evaluate the study outcomes (CLBP pain intensity and disability) and variables of interest (weekly exercise time, daily sitting time, and psychological comorbidities (anxiety and depression) were extracted from the digital self-assessment, which included PHQ-4 sum scores for anxiety and depression [50] and self-report assessments of general health status (first item of the SF-12 questionnaire). Participants were asked to report their current health status so that the insurer could determine the best type of intervention for them and identify individual changes over time. Completion of the digital self-assessment was mandatory for enrolment in the CLBP health intervention.

### Variables

Using binomial and multinomial logistic regression models, we calculated the unadjusted and adjusted odds ratios (OR), with 95% confidence intervals (CI), to determine how well the three variables of interest – exercise (exercise time per week), sedentary behaviour (sitting time per week) and mental health factors (PHQ-4) – predicted CLBP pain intensity and disability. Based on the collected data and known risk factors for CLBP [8, 9], the following variables were treated as potential confounders: overall health status, age, sex, days of exercise, days of sweating, job status, and administrative information indicating the severity of CLBP (total health costs, back pain-specific costs, number of back pain-related ICD-10 diagnoses [“M diagnoses”], and comorbidities). The time of enrolment relative to the pandemic (before or during COVID-19) was treated as a potential effect modifier. The methods for defining and operationalising the study variables are explained in detail below.

### Outcomes: CLBP pain intensity and disability

Von Korff’s Graded Chronic Pain Scale (GCPS) [23, 49], a widely used, frequently validated, and internationally recognised tool for self-administered pain assessment [25, 51–56] was used to measure pain intensity and disability associated with CLBP in the present study. It was included in the digital self-assessment process required for enrolment in the CLPB intervention. The GCPS questionnaire uses three items, rated on a scale of 0 to 10, to obtain a pain intensity score, calculated as the average of 0 to 10 ratings of “pain right now”, “average pain” and “worst pain” multiplied by ten to yield a score of 0 to 100. Participants were assigned to the following pain intensity groups: *No pain* (= 0), *low pain* intensity (< 50) and *high pain* intensity (> = 50). Similarly, the GCPS uses four items to measure disability in terms of the impact of a disease on daily, social and work activities (disability score) and the number of disability days. A disability score is calculated in the same way as the pain intensity score (0–100). Additionally, disability days and score are categorised into groups and combined to total disability points. As the GCPS is formed from combining the pain intensity score and the disability points, the disability points were used in the further analysis. Because persons with 0 disability points can have up to 6 disability days and a disability score of 0 to 29 points, we omitted the *no disability* category and grouped disability as follows: *low disability* (0 disability points), *moderate disability* (< 3 disability points) and *high disability* (≥ 3). Based on their answers to the seven test items, each participant was assigned an overall GCPS grade as follows: Grade 0– no pain problems, Grade I – low to moderate disability, low intensity, Grade II – low to moderate disability, high intensity, Grade III – high disability, moderately limiting, and Grade IV – high disability, severely limiting [23].

### Predictor variables

We hypothesized that the target lifestyle factors (regular exercise and sedentary behaviour) and emotional factors (anxiety and depression) can influence and predict the target outcomes, CLBP-related pain intensity and disability. To test this hypothesis, we extracted data on the presumed predictor variables from the digital self-assessment completed by all participants. It measured regular exercise and sedentary behaviour using items from the German Health Interview and Examination Survey for Adults (DEGS1), an instrument developed by the Robert Koch Institute (Berlin, Germany) [57]. Accordingly, physical activity was operationalised as the amount of exercise time per week. Participants were asked “*How often do you exercise?” and were provided the following* response categories: “l*ess than 1 h ('no sports*')”, “*regularly, 1 to 2 h*”, “*regularly, 2 to 4 h*”, and “*regularly, more than 4 h*” (per week) [57]. Sedentary behaviour was operationalised as time spent sitting per day, which was captured using the question: “*How much time do you spend sitting*?” (percentage of day). Response categories were: “little time”, “about 25%”, “about 50%”, “about 75%”, “almost all day”. Depression and anxiety were measured using the Patient Health Questionnaire 4 (PHQ-4) and its subscales [50]. The PHQ-4 is a four-item questionnaire with answers graded on a four-point scale (0–3), which are summed to yield the total score (0–12). The higher the score, the more likely the presence of depression or anxiety [50].

### Confounders

Other known risk factors for CLBP [8, 9] that could affect the predictor variables were included as potential confounders (disjunctive cause criterion [58]) if they were also captured in the data sets. The following confounders were captured in the digital self-assessment: general health status, age, sex, days, and length of general physical activity. Those extracted from the administrative/insurance database were: job status, variables related to CLBP severity (total health costs, back pain-specific costs, frequency of medical consultation), comorbidities and number of medical consultations for anxiety and depression.

The participants’ general health status was assessed using the first item of the German version of the Short Form 12 (SF-12), a widely used instrument for assessing self-reported health-related quality of life [59]. General exercise status (days and length) was measured by two digital self-assessment questions: 1) “*How many days a week are you physically active enough to start to sweat or get out of breath in an average week?”* (0–7) and 2) “How *long are you physically active on average on days when you start to sweat or get out of breath due to your physical activity?”.* Responses to both questions were: “less than 10”, “10 to less than 30", "30 to less than 60" or "more than 60" (minutes).

Data on health-care costs (general and back pain-specific) and health system utilisation in the last 12 months prior to enrolment were extracted from the insurance database to verify the self-reported data and to obtain more detailed information on the participants’ overall and back pain-specific health status. Costs from the following areas were included: General hospital services, general practitioner and specialist care, medicines, remedies, alternative practitioners (e.g., chiropractors), aids, and private medical treatment. Additional elective services (e.g., one- or two-bed hospital room) and dental treatment costs were excluded. Due to different reimbursement payments under different tariffs, cost components (total and back pain-specific) were defined as the total amount billed (actual costs) instead of the amount reimbursed. As the costs were summed over a period of 12 months, no discounting was performed. All costs were converted to 2020 euro values (€) using consumer price indices.

The frequency of health system utilisation due to back pain-related disorders, defined as ICD-10 codes M40-M54 [“M diagnoses”], and anxiety or depression-related disorders, defined as ICD-10 F* codes F32*, F33*, F34.1, F34.8, F34.9, F38, F41.2, F45.4, F48.0, F43.20, F43.21, F43.22, F54, F62.80 (“F diagnoses”) [60], was calculated as the number of these codes billed during the last 12 months prior to enrolment. The severity of comorbidities in the last 12 months was assessed using the weighted Charlson's Comorbidity Index (CCI) [61]. CCI scores were calculated with the R-package by Gasparini et al. [62].

### Effect modifier

As the influence of the pandemic on the severity of CLBP was the topic of this research, we treated pandemic status as a potential effect modifier. Therefore, participants were stratified into pre-pandemic (*n* = 2,088) and pandemic groups (*n* = 1,390). Also, because of reported differences in pain perception between the sexes [63], men (*n* = 2,288) and women (*n* = 1,190) were examined together and separately.

### Bias

This study may be subject to three sources of bias: recall bias, instrument bias and selection bias. A standardised digital self-assessment procedure was used to collect the data used for assessment of physical activity, sedentary behaviour, health conditions and behaviour patterns. The questionnaire used to assess CLBP pain intensity and disability is an internationally recognised instrument [12] that has been validated several times [10, 11]. However, self-reporting (instrument bias) without objective measures (e.g., activity monitors) could have biased the exercise and sitting time measurements. A lack of agreement between objective and self-reported sitting time has been reported [64]. However, the present study did not resort to precise data on sitting time, but instead asked for general trends and assessments. Furthermore, the accuracy of activity trackers was shown to vary when used to measure daily energy expenditure [65]. It can be assumed that a 5-point cut-off adapted from a Likert scale could accurately depict a trend. Even if this did not allow us to objectively determine the number of hours sitting, it was an indicator of the general effect of sitting time on pain intensity and disability. The assessment of general trends in exercise behaviour also reduced the potential for recall bias. The self-assessment procedure did not capture any exact behaviour at a specific time, but typical behaviour in an average week or on a normal day. This helped to overcome recall bias [66]. Obviously, additional objective measurement of physical activity would have provided the most complete information on active and sedentary behaviour patterns [67]. However, we assume that it is acceptable to use survey instruments with validated questions to map general trends related to the research questions at hand. Last but not least, the lowering of entrance barriers for enrolment in the intervention during the pandemic may have led to selection bias. Subgroup and sensitivity analyses were performed to reduce the potential for selection bias.

### Statistical methods

Patient characteristics were described in terms of frequencies, proportions or means with standard deviation for the overall group and stratified according to sex. Bi- and multinomial logistic regression models were used to calculate the unadjusted and adjusted odds ratio (OR), with 95% confidence interval (CI), to describe the strength of association between the variables of interest and the outcomes (CLBP-related pain intensity and disability). The correlation of possible confounders was checked. If positive or negative correlation was above 0.5, one of the two was included in the model. The unadjusted (predictors only) and adjusted estimates (predictors and selected confounders) are reported in the results section. Model quality was evaluated with Nagelkerkes R2 (acceptable above 0.2) and Hosmer-and-Lemeshow goodness-of-fit tests (*p* > 0.05). To exclude overfitting of the models, the separability of covariance was examined with likelihood ratio tests (*p* < 0.05) and Wald statistics (*p* < 0.05) [68]. The variation inflation factor (VIF) was estimated to test for multicollinearity [69]. As VIF was less than 3 and tolerance greater than 0.3, multicollinearity was not an issue. Subgroup analysis was performed by stratifying the population into “pre-pandemic” and “during the pandemic” groups. A sensitivity analysis was performed to detect potential bias of participant selection for the CLBP intervention. Data management and statistical analyses were performed using R software [70] and the related packages [71–80].

## Results

### Participant characteristics

Data sets from a total of 3,629 participants were identified. Privately insured persons often have an individual annual cost threshold (deductible). Insurees with a fixed deductible usually only submit invoices for reimbursement if they exceed this threshold during the year. To reduce the potential for bias from this, 123 participants who did not provide an invoice in the 12 months before enrolment (annual average invoices = 27) were excluded. Another 28 participants with no pain (GCPS Grade 0) were excluded because CLBP was unlikely, leaving a final study population of 3,478 participants.

The characteristics of the 3,478 participants (mean age: 54.71 years), stratified according to enrolment date as “pre-pandemic” (*n* = 2,088) and “pandemic” (*n* = 1,390), are presented in Table 1. Their self-reported general health was mostly *good* (52.3%) to *very good* (15.7%). Their weighted CCI scores were 1.11 (pre-pandemic) and 0.33 (pandemic). Overall, the total annual health care costs were € 7,280.52. More than two-thirds of the participants were categorised as having GCPS grade I (42.3%) or grade II pain (24.9%), equivalent to low disabling chronic pain.

**Table 1:** Characteristics of study participants according to pandemic status

	***Overall***	***Before COVID-19***	***During COVID-19***	***p***
*Participants (N)*	3,478	2,088	1,390	
*Age (mean (SD))*	54.71 (9.52)	55.80 (8.83)	53.07 (10.26)	< 0.001
*Sex (%)*				< 0.001
* Male*	*2288 (65.8)*	1433 (68.6)	855 (61.5)	
* Female*	*1190 (34.2)*	655 (31.4)	535 (38.5)	
*General health (%)*				< 0.001
* Very good*	545 (15.7)	189 (9.1)	356 (25.6)	
* Good*	1814 (52.2)	1072 (50.8)	754 (54.2)	
* Bad*	1119 (32.2)	839 (40.2)	280 (20.1)	
* Total Health Cost € (mean (SD))*	7280.52 (8033.50)	8805.30 (9126.79)	4990.06 (5258.27)	< 0.001
* Charlons’s Comorbidity Index score – score (mean (SD))*	0.80 (1.41)	1.11 (1.58)	0.33 (0.92)	< 0.001
*GCPS (%)*				< 0.001
* 1*	1471 (42.3)	774 (37.1)	697 (50.1)	
* 2*	867 (24.9)	463 (22.2)	404 (29.1)	
* 3*	622 (17.9)	435 (20.8)	187 (13.5)	
* 4*	518 (14.9)	416 (19.9)	102 (7.3)	
*Disability due to CLBP (%)*				< 0.001
* Low disability*	1197 (34.4)	558 (26.7)	639 (46.0)	
* Moderate disability*	1135 (32.6)	673 (32.2)	462 (33.2)	
* High disability*	1146 (32.9)	857 (41.0)	289 (20.8)	
*Pain intensity due to CLBP (%)*				< 0.001
* Low pain intensity*	1619 (46.5)	888 (42.5)	731 (52.6)	
* High pain intensity*	1859 (53.5)	1200 (57.5)	659 (47.4)	
* Amount of ICD-10 M diagnoses (mean (SD))*	4.13 (5.68)	5.09 (6.58)	2.68 (3.49)	< 0.001
* Total Cost of back pain € (mean (SD))*	1082.93 (2,292.22)	1374.17 (2,779.35)	645.43 (1107.72)	< 0.001
* Amount of ICD-10 F diagnoses (mean (SD))*	1.42 (4.84)	2.10 (5.92)	0.38 (2.06)	< 0.001
* PHQ-4 sum score (mean (SD))*	2.84 (2.56)	3.00 (2.67)	2.59 (2.36)	< 0.001
* PHQ-4 subscale depression*	1.57 (1.41)	1.64 (1.46)	1.46 (1.32)	< 0.001
* PHQ-4 subscale anxiety*	1.27 (1.37)	1.36 (1.42)	1.13 (1.28)	< 0.001
*Job status (%)*				< 0.001
* Not employed*	212 (6.1)	107 (5.1)	105 (7.6)	
* Self employed*	933 (26.8)	645 (30.9)	288 (20.7)	
* Regularly employed*	2333 (67.1)	1336 (64.0)	997 (71.7)	
*Daily sitting time (%)*				0.004
* Less than half of the day*	688 (19.8)	427 (20.5)	261 (18.8)	
* Half of the day*	1008 (29.0)	643 (30.8)	365 (26.3)	
* At least 3/4 of the day*	1166 (33.5)	663 (31.8)	503 (36.2)	
* Almost all day*	616 (17.7)	355 (17.0)	261 (18.8)	
*Activity days per week (%)*				< 0.001
* Never*	474 (13.6)	325 (15.6)	149 (10.7)	
* 1 day*	691 (19.9)	434 (20.8)	257 (18.5)	
* 2 days*	926 (26.6)	545 (26.1)	381 (27.4)	
* 3 days*	705 (20.3)	421 (20.2)	284 (20.4)	
* 4 or more days*	682 (19.6)	363 (17.4)	319 (22.9)	
*Duration physical activity (%)*				< 0.001
* Less than 10 min*	409 (11.8)	272 (13.0)	137 (9.9)	
* 10 to 30 min*	623 (17.9)	386 (18.5)	237 (17.1)	
* 30 to 60 min*	1446 (41.6)	784 (37.5)	662 (47.6)	
* More than 60 min*	1000 (28.8)	646 (30.9)	354 (25.5)	
*Sports per week (%)*				0.001
* No sports*	576 (16.6)	427 (20.5)	149 (10.7)	
* Regularly up to 2 h*	1029 (29.6)	614 (29.4)	415 (29.9)	
* Regularly up to 4 h*	1230 (35.4)	704 (33.7)	526 (37.8)	
* Regularly more than 4 h*	643 (18.5)	343 (16.4)	300 (21.6)	

The number of ICD-10 M40-M54 diagnoses in the last 12 months was more than twice as high in the pandemic group than in the pre-pandemic group (5.09 vs. 2.68, *p* < 0.001). The number of ICD-10 F diagnoses also differed significantly between the two groups (2.1 vs. 0.38, *p* < 0.001). Pre-pandemic participants had more physician visits due to these diagnoses: pre-pandemic participants reported significantly higher levels of anxiety (1.36 vs. 1.13, *p* < 0.001) and depression (1.64 vs. 1.46, *p* < 0.002), as measured on the associated PHQ-4 subscales.

Daily activity also differed significantly between the two groups. Compared to the pre-pandemic group, pandemic participants reported longer sitting times, but they also had, on average, more days of the week with exercise-induced perspiration and more cumulative exercise time per week. In general, participants enrolled during the COVID-19 pandemic were younger and healthier and had lower pain and disability and fewer physician visits due to CLBP.

### CLBP-related disability

As differences in pain perception between the sexes have been previously identified [63], men and women were analysed together and separately. First, we examined the effect of the predictor variables on CLBP-related disability. Two models were run to estimate the associations. Model 1 was not adjusted for confounders. Model 2 consisted of the three predictors and the following potential confounders: age, sex, general health status, Charlson's comorbidity index, general activity days per week, general physical activity duration, number of M diagnoses, number of F diagnoses and job status. Associations detected in Model 2 are described in the text. Three disability categories were defined: *low disability* (34.4%), *moderate disability* (32.6%) and *high disability* (32.9%). The groups were approximately evenly populated. The baseline comparison group consisted of participants with low disability. There were no significant differences in distribution of the disability groups between the two sexes (*p* = 0.963). The association between CLBP disability and the predictors (regular exercise, sedentary behaviour and anxiety/depression) is shown in Table 2. The full model is shown in the appendix (Appendix 1: Pain Disability). After adjusting for confounders, regular exercise was significantly associated with lower odds of high disability in men and women. In particular, four or more hours of regular exercise per week significantly reduced the odds of high disability for men (OR 0.49, 95% CI 0.31 – 0.79, *p* = 0.003) and women (OR 0.30, 95% CI 0.14 – 0.63, *p* = 0.002) compared to the control group that did not exercise. Conversely, high daily sitting times had a positive effect on CLBP disability in men: sitting for at least 75% of the day significantly reduced the odds of high disability in men (OR 0.66, 95% CI 0.46 – 0.95, *p* = 0.025) compared to sitting less than half the day.

**Table 2:** Associations of disability due to CLBP with weekly sports, daily sitting time, and mental health

Moderate Disability	Overall (*n* = 3,478)		Men (*n* = 2,288)		Women (*n* = 1,190)	
	Unadjusted OR (95% CI)	Adjusted OR (95% CI)	Unadjusted OR (95% CI)	Adjusted OR (95% CI)	Unadjusted OR (95% CI)	Adjusted OR (95% CI)
Regular exercise						
No exercise	Reference	Reference	Reference	Reference	Reference	Reference
2–4 h/week	0.72 ^*^ (0.55, 0.94)	0.90 (0.65, 1.23)	0.75 ^+^ (0.54, 1.05)	0.88 (0.60, 1.28)	0.67 (0.41, 1.10)	0.97 (0.55, 1.74)
> 4 h/week	0.46 *** (0.34, 0.62)	0.56 ** (0.39, 0.80)	0.48 *** (0.33, 0.69)	0.55 ** (0.36, 0.85)	0.41** (0.24, 0.73)	0.59 (0.30, 1.16)
Sitting						
Less than half of the day	Reference	Reference	Reference	Reference	Reference	Reference
At least ¾ of the day	1.04 (0.82, 1.32)	1.05 (0.82, 1.36)	1.01 (0.74, 1.38)	1.06 (0.76, 1.47)	0.95 (0.64, 1.41)	0.97 (0.64 -1.49)
Almost all day	0.89 (0.68, 1.18)	0.96 (0.71, 1.29)	0.78 (0.54, 1.11)	0.89 (0.61, 1.29)	1.07 (0.67, 1.71)	1.19 (0.72, 1.97)
Mental health						
PHQ-4 sum score	1.30 *** (1.25, 1.36)	1.23 *** (1.17, 1.29)	1.34 *** (1.27, 1.42)	1.26 *** (1.18, 1.34)	1.25 *** (1.16, 1.35)	1.18*** (1.09, 1.28)
**High Disability**	**Overall (***n*** = 3,478)**		**Men (***n*** = 2,288)**		**Women (***n*** = 1,190)**	
	Unadjusted OR (95% CI)	Adjusted OR (95% CI)	Unadjusted OR (95% CI)	Adjusted OR (95% CI)	Unadjusted OR (95% CI)	Adjusted OR (95% CI)
Regular exercise						
No exercise	Reference	Reference	Reference	Reference	Reference	Reference
2–4 h/week	0.42 ^***^ (0.32, 0.56)	0.61 ^**^ (0.44, 0.86)	0.45 ^***^ (0.32, 0.64)	0.68 ^+^ (0.45, 1.03)	0.39 ^***^ (0.23, 0.64)	0.47 ^*^ (0.25, 0.88)
> 4 h/week	0.34 ^***^ (0.25, 0.47)	0.42 ^***^ (0.28, 0.63)	0.37 ^***^ (0.25, 0.54)	0.49 ** (0.31, 0.79)	0.29 ^***^ (0.16, 0.51)	0.30 ^**^ (0.14, 0.63)
Sitting						
Less than half of the day	Reference	Reference	Reference	Reference	Reference	Reference
At least 3/4 of the day	0.64 ^***^ (0.50, 0.83)	0.70 ^*^ (0.53, 0.93)	0.61 (0.44, 0.84)	0.66 ^*^ (0.46, 0.95)	0.62 ^*^ (0.41, 0.94)	0.73 (0.45, 1.19)
Almost all day	0.64 ^**^ (0.47, 0.86)	0.80 (0.58, 1.12)	0.55 (0.38, 0.81)	0.71 (0.47, 1.07)	0.73 (0.44, 1.21)	1.00 (0.56, 1.79)
Mental health						
PHQ-4 sum score	1.66 ^***^ (1.58, 1.74)	1.50 ^***^ (1.43, 1.59)	1.70 ^***^ (1.60, 1.81)	1.55 ^***^ (1.45, 1.66)	1.62 ^***^ (1.5, 1.75)	1.44 ^***^ (1.32, 1.56)

Model 1: Unadjusted model

Model 2: Adjusted for age, sex, general health status, Charlson's comorbidity index, activity days per week, general physical activity duration, number of M diagnoses, number of F diagnoses, job status

*CI* Confidence interval, *CLBP* Chronic lower back pain, *OR* Odds ratio

^+^  < 0.1

^*^ < 0.05

^**^ < 0.01

^***^ < 0.001

The likelihood of moderate and high disability was also significantly associated with mental health factors (anxiety and depression). A one-point PHQ-4 score rise increased the probability of moderate (OR 1.23, 95% CI 1.17 – 1.29, *p* < 0.001) and high disability (OR 1.50, 95% CI 1.43 – 1.59, *p* < 0.001).

### CLBP pain intensity

The associations between CLBP intensity and weekly exercise time, daily sitting time and mental health factors are shown in Table 3. The full model is shown in the appendix (Appendix 2: Pain Intensity). After adjusting for confounders, four or more hours of regular weekly exercise reduced the odds of high pain intensity in women significantly (OR 0.37, 95% CI 0.21 – 0.65, *p* <  = 0.001) compared to the odds in women who did not exercise (reference). No association between pain intensity and daily sitting time was observed in men or in women. Psychological factors (anxiety and depression) were significantly associated with high intensity pain in both sexes. In the overall model, each one-point increase in PHQ-4 score was associated with an increased probability of having high pain intensity (OR 1.27, CI 1.22 – 1.32, *p* < 0.001).

**Table 3:** Associations of high low back pain intensity with weekly exercise time, daily sitting time, and mental health factors (anxiety and depression)

High pain intensity	Overall (*n* = 3,478)		Men (*n* = 2,288)		Women (*n* = 1,190)	
	*Unadjusted* *OR (95% CI)*	*Adjusted* *OR (95% CI)*	*Unadjusted* *OR (95% CI)*	*Adjusted* *OR (95% CI)*	*Unadjusted* *OR (95% CI)*	*Adjusted* *OR (95% CI)*
*Regular exercise*						
No exercise	Reference	Reference	Reference	Reference	Reference	Reference
2–4 h/week	0.68 ^***^ (0.55, 0.85)	0.89 (0.69, 1.15)	0.69 ^**^ (0.53, 0.90)	0.97 (0.71, 1.32)	0.63 ^*^ (0.42, 0.94)	0.71 (0.44, 1.14)
> 4 h/week	0.58 ^***^ (0.45, 0.74)	0.66 ^**^ (0.49, 0.89)	0.67 ^**^ (0.50, 0.90)	0.85 (0.59, 1.21)	0.41 ^***^ (0.25, 0.64)	0.37 ^***^ (0.21, 0.65)
*Sitting*						
Less than half of the day	Reference	Reference	Reference	Reference	Reference	Reference
At least 3/4 of the day	0.90 (0.73, 1.10)	0.99 (0.80, 1.23)	0.94 (0.73, 1.22)	1.02 (0.77, 1.35)	0.84 (0.60, 1.17)	0.90 (0.62, 1.28)
Almost all day	0.83 (0.66, 1.05)	0.96 (0.74, 1.23)	0.82 (0.61, 1.10)	0.94 (0.68, 1.29)	0.88 (0.59, 1.31)	1.01 (0.66, 1.55)
*Mental health*						
PHQ-4 sum score	1.36 ^***^ (1.32, 1.41)	1.27 ^***^ (1.22, 1.32)	1.39 ^***^ (1.33, 1.45)	1.28 ^***^ (1.22, 1.34)	1.32 ^***^ (1.25, 1.40)	1.25 ^***^ (1.18, 1.34)

Model 1: Unadjusted model

Model 2: Adjusted for age, sex, general health status, Charlson's comorbidity index, activity days per week, general physical activity duration, number of M diagnoses, number of F diagnoses, job status

*CI* Confidence interval, *CLBP* Chronic lower back pain, *OR* Odds ratio

^+^  < 0.1

^*^ < 0.05

^**^ < 0.01

^***^ < 0.001

### Subgroup analysis

As changes due to the COVID-19 pandemic could influence risk factors for CLBP intensity and disability, pre-pandemic (*n* = 2,088) and pandemic group (*n* = 1,390) were analysed separately. The observed associations of CLBP disability and pain intensity with cumulative weekly exercise time, daily sitting time and mental health factors are presented in Table 4. Presented are the results for the adjusted model (Model 2) for disability and pain intensity, which was applied to both subgroups. The full model is shown in the appendix (Appendix 3: Subgroup analyses). Four or more hours of regular weekly exercise significantly reduced the odds of high disability in the pre-pandemic (OR 0.57, 95% CI 0.36 – 0.92, *p* = 0.021) and pandemic groups (OR 0.29, 95% CI 0.124 – 0.558, *p* = 0.002) compared to the control group (no regular weekly exercise). Two to four hours of regular weekly exercise reduced the odds of high disability in the pandemic group (OR 0.42, 95% CI 0.22 – 0.83, *p* = 0.012) but not in the pre-pandemic group. Neither exercise nor sitting time was significantly associated with moderate disability from CLBP in the pre-pandemic group. However, four or more hours of regular weekly exercise reduced the odds of moderate disability in the pandemic group (OR 0.38, 95% CI 0.20 – 0.71, *p* = 0.002).

**Table 4:** Associations of CLBP pain intensity and disability in the pre-pandemic vs. pandemic group

*Disability*	Pre-pandemic group (*n* = 2,088)		Pandemic group (*n* = 1,390)	
	*Moderate Disability* *OR (95% CI)*	*High Disability* *OR (95% CI)*	*Moderate Disability* *OR (95% CI)*	*High Disability* *OR (95% CI)*
Regular exercise				
* No exercise*	Reference	Reference	Reference	Reference
* 2–4 h/week*	1.13 (0.76, 1.68)	0.80 (0.54, 1.21)	0.62 ^+^ (0.36, 1.08)	0.42 ^*^ (0.22, 0.83)
> *4 h/week*	0.71 (0.45, 1.13)	0.57 ^*^ (0.36, 0.92)	0.38 ^**^ (0.20, 0.71)	0.29 ^**^ (0.13, 0.64)
Sitting				
* Less than half of the day*	Reference	Reference	Reference	Reference
* At least ¾ of the day*	1.00 (0.71, 1.42)	0.77 (0.53, 1.11)	1.18 (0.80, 1.74)	0.63 ^+^ (0.38, 1.03)
* Almost all day*	0.96 (0.64, 1.44)	0.84 (0.55, 1.29)	0.99 (0.63, 1.55)	0.75 (0.43, 1.33)
Mental health				
* PHQ-4 sum score*	1.16 ^***^ (1.08, 1.24)	1.42 ^***^ (1.33, 1.52)	1.35 ^***^ (1.25, 1.46)	1.73 ^***^ (1.58, 1.90)
***Pain Intensity***	*Low intensity* *(base outcome)*	*High intensity* *OR (95% CI)*	*Low intensity* *(base outcome)*	*High intensity* *OR (95% CI)*
Regular exercise				
* No exercise*		Reference		Reference
* 2–4 h/week*		0.91 (0.67, 1.24)		0.87 (0.54, 1.41)
> *4 h/week*		0.65 ^*^ (0.45, 0.94)		0.67 (0.38, 1.17)
Sitting				
* Less than half of the day*		Reference		Reference
* At least ¾ of the day*		0.96 (0.73, 1.27)		1.04 (0.73, 1.48)
* Almost all day*		0.96 (0.69, 1.34)		0.94 (0.63, 1.41)
Mental health				
* PHQ-4 sum score*		1.22 ^***^ (1.16, 1.28)		1.36 ^***^ (1.28, 1.46)

Disability: Adjusted disability model stratified into pre-pandemic vs. pandemic groups

Intensity: Adjusted intensity model stratified into pre-pandemic vs. pandemic groups

*CI* Confidence interval, *CLBP* Chronic lower back pain, *OR* Odds ratio

^+^  < 0.1

^*^ < 0.05

^**^ < 0.01

^***^ < 0.001

The importance of mental health also increased during the pandemic. In the pre-pandemic group, each one-point increase in PHQ-4 score was associated with an increased probability of moderate (OR 1.16, 95% CI 1.08 – 1.24, *p* < 0.001) and high disability (OR 1.42, 95% CI 1.33 – 1.52, *p* < 0.001). In the pandemic group, each marginal increase in PHQ-4 score increased the odds for moderate pain disability to 1.35 (95% CI 1.25 – 1.46, *p* < 0.001) and those for high pain disability to 1.73 (95% CI 1.58 – 1.89, *p* < 0.001).

Sitting time was not significantly associated with CLBP pain intensity in either group. The prevalence of high pain intensity was significantly reduced in pre-pandemic participants who regularly exercised four or more hours/week (OR 0.65 95% CI 0.45 – 0.94, *p* = 0.022). In the pandemic group, however, neither sitting time nor exercise time significantly affected pain intensity. An increased PHQ-4 sum score was significantly associated with an increased probability of high pain intensity in participants enrolled before and during the pandemic. The association was greater in the pandemic group.

### Sensitivity analysis

Participants were enrolled in the intervention by invitation from the insurer and by request (self-selection). Self-selected participants were not checked for administrative indication of CLBP in a standardised manner. The decision to enrol these individuals was based on a telephone assessment by customer service personnel. Self-selected participants’ statements regarding the relevant diagnoses were not checked for accuracy. It is questionable whether all of those individuals actually had CLBP. To rule out the influence of self-selection, we performed a sensitivity analysis that excluded all self-selected participants (*n* = 803). The information presented in Table 5 is the same as in the subgroup analysis, with adapted group sizes. The full model is shown in the appendix (Appendix 4: Sensitivity analyses). The sensitivity analysis included 1,411 participants in the pre-pandemic group and 1,267 participants in the pandemic group. Most associations remained significant and had the same direction of effect, with only slight changes in effect sizes compared to the subgroup analysis. The biggest difference was in the effect of exercise on disability and intensity in the pre-pandemic group. In contrast to the overall subgroup analysis, the sensitivity analysis group indicated that four or more hours of regular exercise/week did not have a significant effect on high disability or high pain intensity in the pre-pandemic group.

**Table 5:** Sensitivity analysis: Associations of CLBP pain intensity and disability in the pre-pandemic vs. pandemic group of invited participants only

*Disability model*	Pre-pandemic group (*n* = 1,411)		Pandemic group (*n* = 1,267)	
	*Moderate disability OR (95% CI)*	*High disability OR (95% CI)*	*Moderate disability OR (95% CI)*	*High disability OR (95% CI)*
*Regular exercise*				
* No exercise*	Reference	Reference	Reference	Reference
* 2–4 h/week*	1.27 (0.79, 2.06)	0.98 (0.60, 1.59)	0.64 (0.36, 1.13)	0.42 * (0.20, 0.86)
> *4 h/week*	0.77 (0.44, 1.35)	0.66 (0.37, 1.18)	0.39 ** (0.20, 0.76)	0.30 ** (0.13, 0.68)
*Sitting*				
* Less than half of the day*	Reference	Reference	Reference	Reference
* At least 3/4 of the day*	0.93 (0.61, 1.41)	0.85 (0.55, 1.32)	1.16 (0.77, 1.74)	0.63 + (0.37, 1.07)
* Almost all day*	0.71 (0.42, 1.17)	0.72 (0.43, 1.22)	0.99 (0.62, 1.58)	0.74 (0.40, 1.35)
*Mental health*				
* PHQ-4 sum score*	1.12 ^**^ (1.03, 1.22)	1.44 ^***^ (1.33, 1.56)	1.33 ^***^ (1.22, 1.44)	1.67 ^***^ (1.52, 1.84)
***Pain intensity model***	*Low intensity* *(base outcome)*	*High intensity* *OR (95% CI)*	*Low intensity* *(base outcome)*	*High intensity* *OR (95% CI)*
*Regular exercise*				
* No exercise*		Reference		Reference
* 2–4 h/week*		1.07 (0.74, 1.55)		0.93 (0.55, 1.56)
> *4 h/week*		0.69 (0.44, 1.07)		0.77 (0.42, 1.40)
*Sitting*				
* Less than half of the day*		Reference		Reference
* At least 3/4 of the day*		1.00 (0.71, 1.40)		0.99 (0.69, 1.43)
* Almost all day*		0.92 (0.61, 1.38)		0.91 (0.59, 1.39
*Mental health*				
* PHQ-4 sum score*		1.24 ^***^ (1.18, 1.32)		1.36 ^***^ (1.27, 1.46)

Disability: Adjusted disability model stratified into pre-pandemic vs. pandemic groups with enrolment by invitation only

Intensity: Adjusted intensity model stratified into pre-pandemic vs. pandemic groups with enrolment by invitation only

*CI* confidence interval, *CLBP* chronic lower back pain, *OR* odds ratio

^+^  < 0.1

^*^ < 0.05

^**^ < 0.01

^***^ < 0.001

## Discussion

### Summary of key results

The present study used insurance claims data and self-reported patient information to investigate associations between regular exercise, sitting time, and mental health factors (anxiety and depression) with CLBP pain intensity and disability in a population of patients in Germany. Additionally, a subgroup analysis was performed to compare participants enrolled before and during the COVID-19 pandemic and verified in a sensitivity analysis.

The results of the analyses with binomial and multinomial regression models showed that four or more hours/week of regular exercise significantly reduced the odds of high disability for men (OR = 0.49) and women (OR = 0.30) compared to non-exercisers. Conversely, increasing sitting time to at least ¾ of the day significantly reduced the odds of high disability in men (OR = 0.66), whereas sitting time had no significant effect on disability in women. Mental health issues (anxiety/depression) were a strong predictor of high disability in men and women. Each one-point increase in PHQ-score increased the odds of high disability (OR = 1.5).

Regarding pain intensity, four or more hours/ week of exercise reduced the probability of severe pain in women (OR = 0.37), but exercise had no effect on pain intensity in men. In neither men nor women did the amount of time spent sitting have a statistically significant effect on pain intensity. The psychological factors anxiety and depression had a significant effect on pain intensity. Each increase in PHQ-4 score increased the odds of high pain intensity by an OR of 1.27.

The subgroup analysis compared participants enrolled before and during the pandemic. Participants enrolled during the pandemic were younger and healthier according to their SF-12 and CCI scores, had lower levels of CLBP pain and disability and had fewer care provider visits and, thus, lower health care costs compared to the pre-pandemic group. The magnitude of association of physical activity and mental health on disability and pain intensity increased. In individuals who exercised four hours or more per week, the OR of high disability was 0.57 in the pre-pandemic group compared to 0.29 in the pandemic group. The impact of mental health factors (anxiety and depression) on the odds of high disability also increased: The OR for high disability was 1.42 per marginal increase in the PHQ-4 scale before the pandemic, and 1.73 during the pandemic. Sensitivity analyses of participants with administratively verified CLBP further emphasized the importance of regular weekly exercise during the pandemic.

### Interpretation

The results showed that at least four hours a regular exercise per week in leisure time significantly reduced the odds of high disability in patients with CLBP. This is in line with all clinical guidelines for the management of chronic non‑specific low back pain [81] and clinical experience. Regular exercise significantly increases the chances of having no or low disability due to CLBP [82]. The findings of the systematic review by Lin et al. point in the same direction, showing that higher levels of disability correlate with lower levels of physical activity [83]. Furthermore, Hayden et al. showed in a recently published Cochrane review that exercise improves pain and functional limitations in patients with CLBP compared to usual care and other conservative treatments in the short and medium term [84].

Interestingly, the present study revealed that being sedentary (sitting) for at least 3/4 of the day reduced disability related to high levels of back pain in men. However, the time spent sitting had no effect on pain intensity. Generally, the association between sitting time and CLBP intensity/disability is not widely studied and, if studied, the relationship between sitting and LBP is usually the focus of interest [17]. Studies suggest that although sitting does not improve CLBP pain [85], it tends to allow the affected individuals to perform their daily activities [86, 87]. Sitting is a particular risk factor when associated with whole-body vibrations (e.g., driving a truck or bus) or awkward body positions [17, 88]. We assume that the probability of inclusion of subjects with these characteristics in the sample as low since persons with private health insurance usually belong to higher socioeconomic classes [38, 39]. Similarly, Lunde et al. observed a protective effect of sitting for healthcare workers and suggested that this might be due to sedentary jobs providing higher levels of control and greater autonomy [89]. Furthermore, increased sitting means less time spent standing, which can be a risk factor on its own [90]. Moreover, the conclusions drawn in the present study are based on subjectively measures (self-reports). The results could differ if sitting was evaluated using objective measures [21].

Apart from the protective effect of increased sitting in men, other gender differences were observed in our study. The protective effect of exercise was greater in women than in men, and four or more hours/week of regular exercise was associated with a decrease in pain intensity in women only. We were unable to identify any gender-specific characteristics in the data that could explain these differences. Hussain et al. suggested differences in muscle structure and postural alignment in men and women as a reason [91]. According to the theory of the U-shaped relationship between exercise and CLBP [92], it is possible that over-training in male participants may have produced an adverse effect. As there was no upper limit on the reported exercise time, this could be a factor.

In the present study, the effect of the mental health factors anxiety and depression was evident. Each increase in the PHQ-4 score resulted in significantly higher odds of high disability and high pain intensity. This fits well with current knowledge [93, 94]. Stevans et al. [95] identified depression or anxiety disorders as significant risk factors for the transition from acute to chronic back pain in 5,233 patients with acute LBP. Oliveira et al. also identified greater severity of pain and greater pain-related disability in CLBP patients with anxiety and depression, as determined based on ICD-10 F diagnoses [96]. Our finding that an increase in the number of F* diagnoses was associated with a slight decrease in the odds of disability and high pain intensity suggests that prior treatment of these mental health disorders might increase the success of CLBP treatment (see Appendix 1: Pain Disability, Appendix 2: Pain Intensity). Overall, mental health significantly influenced the relationship between pain intensity and disability. Therefore, the importance of mental health should not be underestimated in the treatment of CLBP. As shown by Hayden et al., an addition of co-interventions (like cognitive behavioural therapy or pain neuroscience education) can improve the outcome of physical treatments for patients with CLBP [97].

A key new finding of the present study is the effect of COVID-19-induced lockdowns on risk factors. We showed that the strength of the association between the variables of interest and high CLBP pain intensity and disability increased during the pandemic. Both the protective effect of exercise and the negative association due to the presence of anxiety or depression were more pronounced in participants surveyed during the lockdown. This does not bode well of CLBP sufferers. Globally, levels of stress, anxiety, and depression have increased due to the uncertainties and consequences of the pandemic [4]. At the same time, a significant decrease in physical activity has been observed [1]. Therefore, it is to be feared that the prevalence of high disability and high intensity due to CLBP will increase in the coming months and years. Although significant decreases in CLBP-related health care system use were observed in 2020, an increase in the level after the pandemic seems conceivable [10]. Our study also revealed a significant decrease in doctor visits due to CLBP during the pandemic compared to pre-pandemic levels, resulting in a 50% reduction of back pain-specific costs. One explanation for this could be the avoidance of contacts during lockdowns [4, 11]. The predominantly digital approach of the CLBP health programme from the beginning of the pandemic may also have contributed to this. Additionally, entrance barriers to the intervention were lowered with the start of the pandemic, and a slightly different subgroup took advantage of the programme. However, a previous study indicated that doctor visits perceived as non-essential were postponed during the lockdown [98].

The protective effect of exercise could be related to the total amount of movement. People who managed to exercise regularly, even in a lockdown situation, might have maintained their level of movement despite having limited opportunities to exercise in daily life.

The association of anxiety or depression with high pain disability and intensity was more pronounced during the lockdown. This effect could be related to the fact that worry, fear, and uncertainty about the future were greater than ever [99]. Many self-employed, usually privately insured persons in the retail and service sector faced greater uncertainties due to the lockdown [100]. More in-depth studies are needed to explain the effect of lockdown-induced exercise reduction and the mechanisms behind the increase in anxiety and depression during the pandemic.

### Strengths, limitations and generalisability

Limitations of this study related to the type of data used (I), the data collection methods (II), and the study design (III). Part of the data was extracted from a private health insurance payer database intended not primarily for research, but for the settlement of claims and for management of their CLBP intervention. Persons with private health insurance generally have a higher socioeconomic status with better health risks [38]. If replicated using statutory health insurance data reflecting the full spectrum of social classes, different effects of the predictors might be found. Additionally, a diagnosis of CLBP was assumed based on insurance claims data in the case of invited participants, and based on the patient’s response to a specific screening question in the case of self-selected participants. Therefore, we cannot definitively confirm that all participants included in the study had CLBP. Our confidence in the administrative selection process is fairly high, but self-reported responses to screening questions about the duration of back pain via the phone have a great margin of error. To control for this potential bias, we performed a sensitivity analysis excluding all self-selected participants, which produced comparable associations.

The results of this study must be interpreted cautiously due to differences in participant characteristics between the pre-pandemic and pandemic period. Participants enrolled during the lockdown were younger, healthier, had lower levels of pain and disability due to CLBP, did not visit the physicians as often and had a lower number of F diagnoses. This could possibly be attributed to the different types of interventions (on-site vs. digital) and entrance barriers that were present in the two periods. It is possible that the different baseline characteristics influenced the relationship between exercise, sedentary behaviour and CLBP. Due to the lockdown situation, this could not be mitigated, but should be closely monitored when both types of intervention are completely accessible again.

A further limitation of the data used is that the participants were not stratified by the number of treatments received. It remains unclear how much CLBP treatment they received before enrolment in the intervention. All invited participants needed a minimum number of diagnoses in a certain time span to receive an invitation, but no maximum treatment period or dose was specified. Therefore, the representativeness of the results is limited, as different participants could have answered the assessment questionnaires at different points in their disease trajectory.

Since we did not have access to data on all factors that could potentially influence CLBP severity (e.g., cognitive factors, social factors, and coping responses), they could not be analysed as potential confounders. Therefore, overestimation of the effects of the predictors evaluated in this study is possible.

The further limitation was the method of collecting data on exercise and sedentary behaviour. Although the questionnaires were validated and used by large health institutions (e.g., the Robert Koch-Institute (RKI), the German institute for disease control and prevention), objective measurement of exercise and sedentary behaviour could produce different results. Self-reported sitting time has low to moderate validity [21] and can underestimate total sitting time [64]. However, objective measurement was not possible here because of the cross-sectional study design. As more than 3,500 participants were enrolled in this study, it was decided to use short questionnaires to keep time constraints and resources feasible. Bakker et al. showed that short questionnaires showed validity and reliability similar to longer questionnaires, so that for this study design, the type of data collection was acceptable [101]. Data collection was carried out at the beginning of the CLBP intervention. This meant that a large number of respondents could be considered, but objective information on exercise behaviour could not be used before the start of the intervention. This is another limitation. Due to the cross-sectional study design, causal relationships could not be established. Therefore, the results should be interpreted cautiously and the possibility of reverse causality should be kept in mind. Future research linking additional objective measurements of exercise and sedentary behaviour at multiple survey time points are needed to improve the results.

At the same time, the study had several strengths that allow for a certain generalisability of the results. A study population of 3,478 participants can generate statistically valid results. Our pre-pandemic and pandemic participants were recruited from the same insurance provider, so their backgrounds and characteristics were highly comparable. They answered the same validated questionnaires, which is why the responses could be interpreted in the same way.

Both real-world evidence (claims data) and validated survey instruments (self-assessment questionnaires) were combined. This combination has been called the most powerful evidence-based research method in medicine [108]. In most studies on physical activity and CLBP, physical activity is defined as the sum of all leisure and occupational activities, so the effect of any individual type of activity is difficult to distinguish [87]. In our regression model selection process, daily physical activity and exercise-specific activity were assessed separately to enable a distinction between daily and exercise-specific movement. The results of the study were very clear and provide important information for CLBP treatment. The study was set in Germany, but many countries were in a similar lockdown situation, so the results can be transferred and generalised to other contexts.

## Conclusions

We analysed the association of exercise, sedentary behaviour and mental health on pain intensity and disability in population of 3,478 participants of a health programme for CLBP patients. At least four hours a week of regular exercise significantly reduced the odds of high disability in all patients with CLBP. The protective effect of exercise was greater in women than in men. Four or more hours a week of regular exercise was associated with a decrease in pain intensity in women only. Sedentary behaviour had no significant effect on pain intensity in men or women. The effect of the mental health factors anxiety and depression on the severity of CLBP was evident. Each increase in the PHQ-4 score resulted in significantly higher odds of high disability and high pain intensity. COVID-19-induced lockdowns had a significant effect on the strength of association of the observed variables. Back pain-specific costs were halved. The magnitude of association of the factors that influenced high pain intensity and disability increased during the pandemic. On the one hand, the protective effect of exercise was greater. On the other hand, the odds of high pain intensity and disability associated with the presence of indicators of anxiety or depression were also more pronounced in participants surveyed during the lockdown.

This information could be used by clinicians and decision-makers to promote regular exercise. A screening process for depression and anxiety with an optional referral to a mental health specialist might be recommended. Further research could try to explain the identified gender differences to understand the underlying mechanisms associated with sedentary behaviour and exercise. The results of the study should be further validated in a setting with objective measurement of sedentary behaviour and exercise. More in-depth study of the lockdown-induced interaction between limited daily physical activity, increased levels of anxiety and depression, and its effect on CLPB could also be worthwhile.

## Supplementary Information

**Additional file 1.**

## Data Availability

The data and code that support the findings of this study are available from Generali Deutschland, but restrictions apply to the availability of these data, which were used under a license agreement for the current study and are not publicly available. Due to potentially identifying and sensitive nature of the patient information, the data cannot be shared publicly. The pseudonymized data are, however, available from the authors upon reasonable request and with permission, including a signed data access agreement from Generali Deutschland. Data management and statistical analyses were performed using the software R with the packages listed in the bibliography. The code supporting the findings of this study is available from Generali Deutschland, but restrictions apply. It is available from the authors upon reasonable request and with the necessary permissions, including a signed access agreement from Generali Deutschland.

## Abbreviations

CCI: Charlson's Comorbidity Index
CI: Confidence interval
CLBP: Nonspecific chronic lower back pain
COVID-19: Coronavirus Disease 2019
GCPS: Graded Chronic Pain Scale
ICD-10: 10Th revision of the International Statistical Classification of Diseases and Related Health Problems
MET: Metabolic equivalent
OR: Odds ratio
PHQ-4: Patient Health Questionnaire 4
RKI: Robert Koch Institute
VIF: Variance Inflation Factor
