# Supplementary material for: Association between chronic low back pain and regular exercise, sedentary behaviour and mental health before and during COVID-19 pandemic: insights from a large-scale cross-sectional study in Germany
Source: BMC Musculoskelet Disord. 2022 Sep 15;23:860. doi: 10.1186/s12891-022-05806-8 (PMC9474280; doi:10.1186/s12891-022-05806-8)
Supplement: Supplementary file 1 — Additional file 1. [file 12891_2022_5806_MOESM1_ESM.pdf]

Appendix:

Content:

Appendix 1: Pain Disability **2**

Appendix 1.1 - Adjusted Modell - Pain Disability *2*

Appendix 1.2 - Adjusted Modell - Pain Disability - Sex = Female *3*

Appendix 1.3 - Adjusted Modell - Pain Disability - Sex = Male *2*

Appendix 2: Pain Intensity **3**

Appendix 2.1 - Adjusted Modell - Pain Intensity *3*

Appendix 2.2 - Adjusted Modell - Pain Intensity - Sex = Female *4*

Appendix 2.3 - Adjusted Modell - Pain Intensity - Sex = Male *5*

Appendix 3: Subgroup analyses **6**

Appendix 3.1 - Adjusted Modell - Pain Disability – Pre Covid *6*

Appendix 3.2 - Adjusted Modell - Pain Disability – During Covid *7*

Appendix 3.3 - Adjusted Modell - Pain Intensity – Pre Covid *8*

Appendix 3.4 - Adjusted Modell - Pain Intensity – During Covid *9*

Appendix 4: Sensitivity analyses **10**

Appendix 4.1 - Adjusted Modell - Pain Disability – Pre Covid - Sensitivity *10*

Appendix 4.2 - Adjusted Modell - Pain Disability – During Covid Sensitivity *11*

Appendix 4.3 - Adjusted Modell - Pain Intensity – Pre Covid - Sensitivity *12*

Appendix 4.4 - Adjusted Modell - Pain Intensity – During Covid - Sensitivity *13*

Appendix 1: Pain Disability

Appendix 1.1 - Adjusted Modell - Pain Disability

Appendix 1.1 - Adjusted Modell - Pain Disability

	02_moderate pain disability			03_high pain disability		
Characteristic	OR[1]	95% CI[1]	p-value	OR[1]	95% CI[1]	p-value
Overall health						
01_Very good	—	—		—	—	
02_good	1.82	1.43, 2.31	**<0.001**	2.06	1.49, 2.83	**<0.001**
03_bad	4.97	3.62, 6.82	**<0.001**	10.0	6.89, 14.6	**<0.001**
PHQ-4	1.23	1.17, 1.29	**<0.001**	1.50	1.43, 1.59	**<0.001**
Activity days per week						
00_never	—	—		—	—	
01_1day_per_wk	0.68	0.45, 1.01	**0.057**	0.62	0.40, 0.97	**0.035**
02_2days_per_wk	0.71	0.47, 1.06	**0.090**	0.72	0.46, 1.13	0.15
03_3days_per_wk	0.83	0.55, 1.26	0.39	0.84	0.52, 1.34	0.46
04_four or more	0.72	0.47, 1.11	0.14	1.03	0.64, 1.66	0.90
Sport days per week						
01_no sports	—	—		—	—	
02_less than two h per week	0.90	0.66, 1.23	0.51	0.78	0.57, 1.09	0.14
03_two to 4h per week	0.90	0.65, 1.23	0.50	0.61	0.44, 0.86	**0.005**
04_more than four h per week	0.56	0.39, 0.80	**0.002**	0.42	0.28, 0.63	**<0.001**
Duration physical activity						
01_less than ten minutes	—	—		—	—	
02_10 to 30 minutes	0.94	0.60, 1.46	0.78	1.05	0.65, 1.69	0.85
03_30 to 60 minutes	1.05	0.68, 1.62	0.82	1.03	0.64, 1.65	0.90
04_more than 60 minutes	1.19	0.76, 1.85	0.45	1.06	0.65, 1.73	0.81
Sex						
M	—	—		—	—	
W	0.91	0.75, 1.10	0.32	0.87	0.70, 1.08	0.20
Age	1.00	0.99, 1.01	0.49	1.02	1.01, 1.03	**0.004**
Number of M-Diagnoses	1.07	1.04, 1.09	**<0.001**	1.17	1.14, 1.19	**<0.001**
Number of F-Diagnoses	0.99	0.97, 1.01	0.34	0.99	0.96, 1.01	0.29
Sitting_time						
01_less than half of the day	—	—		—	—	
02_half of the day	1.02	0.79, 1.31	0.90	0.82	0.62, 1.09	0.18
03_at least 3/4 of the day	1.05	0.82, 1.36	0.70	0.70	0.53, 0.93	**0.015**
04_almost all day	0.96	0.71, 1.29	0.79	0.80	0.58, 1.12	0.20
Job Status						
01_unemployed	—	—		—	—	
02_self_employed	1.12	0.75, 1.67	0.59	1.06	0.68, 1.66	0.80
03_regularly_employed	1.11	0.76, 1.61	0.59	0.92	0.60, 1.39	0.68
wscore	0.96	0.90, 1.03	0.30	0.97	0.90, 1.05	0.50

[1]OR = Odds Ratio, CI = Confidence Interval

Appendix 1.2 - Adjusted Modell - Pain Disability - Sex = Female

Appendix 1.2 - Adjusted Modell - Pain Disability - Sex = Female						
	02_moderate pain disability			03_high pain disability		
Characteristic	OR[1]	95% CI[1]	p-value	OR[1]	95% CI[1]	p-value
Overall health						
01_Very good	—	—		—	—	
02_good	1.57	1.04, 2.36	**0.031**	2.08	1.18, 3.67	**0.012**
03_bad	4.46	2.66, 7.50	**<0.001**	10.4	5.49, 19.6	**<0.001**
PHQ-4	1.18	1.09, 1.28	**<0.001**	1.44	1.32, 1.56	**<0.001**
Activity days per week						
00_never	—	—		—	—	
01_1day_per_wk	0.71	0.34, 1.49	0.37	0.69	0.30, 1.59	0.38
02_2days_per_wk	0.44	0.21, 0.94	**0.035**	0.69	0.29, 1.63	0.40
03_3days_per_wk	0.56	0.26, 1.23	0.15	0.79	0.32, 1.93	0.61
04_four or more	0.47	0.21, 1.05	**0.067**	1.17	0.48, 2.84	0.74
Sport days per week						
01_no sports	—	—		—	—	
02_less than two h per week	1.09	0.63, 1.91	0.75	0.81	0.45, 1.47	0.49
03_two to 4h per week	0.97	0.55, 1.74	0.93	0.47	0.25, 0.88	**0.018**
04_more than four h per week	0.59	0.30, 1.16	0.13	0.30	0.14, 0.63	**0.002**
Duration physical activity						
01_less than ten minutes	—	—		—	—	
02_10 to 30 minutes	0.66	0.30, 1.44	0.29	0.76	0.32, 1.83	0.54
03_30 to 60 minutes	1.00	0.47, 2.12	>0.99	1.04	0.44, 2.44	0.93
04_more than 60 minutes	1.28	0.59, 2.78	0.54	1.09	0.45, 2.63	0.85
Age	1.01	0.99, 1.02	0.46	1.02	1.01, 1.04	**0.009**
Number of M-Diagnoses	1.09	1.04, 1.14	**<0.001**	1.20	1.14, 1.26	**<0.001**
Number of F-Diagnoses	1.00	0.97, 1.04	0.89	1.02	0.98, 1.06	0.30
Sitting_time						
01_less than half of the day	—	—		—	—	
02_half of the day	1.21	0.81, 1.82	0.35	0.93	0.58, 1.47	0.75
03_at least 3/4 of the day	0.97	0.64, 1.49	0.90	0.73	0.45, 1.19	0.21
04_almost all day	1.19	0.72, 1.97	0.50	1.00	0.56, 1.79	>0.99
Job Status						
01_unemployed	—	—		—	—	
02_self_employed	1.19	0.68, 2.08	0.55	0.97	0.52, 1.80	0.92
03_regularly_employed	1.04	0.64, 1.70	0.87	0.74	0.43, 1.27	0.27
wscore	0.96	0.84, 1.10	0.58	1.05	0.91, 1.20	0.51

[1]OR = Odds Ratio, CI = Confidence Interval

Appendix 1.3 - Adjusted Modell - Pain Disability - Sex = Male

Appendix 1.3 - Adjusted Modell - Pain Disability - Sex = Male						
	02_moderate pain disability			03_high pain disability		
Characteristic	OR[1]	95% CI[1]	p-value	OR[1]	95% CI[1]	p-value
Overall health						
01_Very good	—	—		—	—	
02_good	1.95	1.45, 2.63	**<0.001**	1.98	1.34, 2.93	**<0.001**
03_bad	5.44	3.63, 8.15	**<0.001**	10.0	6.27, 16.0	**<0.001**
PHQ-4	1.26	1.18, 1.34	**<0.001**	1.55	1.45, 1.66	**<0.001**
Activity days per week						
00_never	—	—		—	—	
01_1day_per_wk	0.60	0.37, 0.99	**0.044**	0.55	0.32, 0.94	**0.029**
02_2days_per_wk	0.83	0.52, 1.35	0.46	0.70	0.41, 1.18	0.18
03_3days_per_wk	0.96	0.58, 1.59	0.88	0.83	0.48, 1.45	0.51
04_four or more	0.85	0.50, 1.43	0.54	0.93	0.53, 1.63	0.79
Sport days per week						
01_no sports	—	—		—	—	
02_less than two h per week	0.83	0.58, 1.21	0.33	0.74	0.50, 1.10	0.14
03_two to 4h per week	0.88	0.60, 1.28	0.50	0.68	0.45, 1.03	**0.068**
04_more than four h per week	0.55	0.36, 0.85	**0.007**	0.49	0.31, 0.79	**0.003**
Duration physical activity						
01_less than ten minutes	—	—		—	—	
02_10 to 30 minutes	1.17	0.68, 2.02	0.57	1.28	0.72, 2.29	0.40
03_30 to 60 minutes	1.15	0.67, 1.97	0.61	1.07	0.60, 1.91	0.81
04_more than 60 minutes	1.21	0.70, 2.09	0.49	1.08	0.60, 1.95	0.79
Age	1.00	0.99, 1.01	0.91	1.01	1.00, 1.03	0.13
Number of M-Diagnoses	1.06	1.03, 1.09	**<0.001**	1.16	1.12, 1.19	**<0.001**
Number of F-Diagnoses	0.98	0.95, 1.01	0.16	0.96	0.93, 0.99	**0.012**
Sitting_time						
01_less than half of the day	—	—		—	—	
02_half of the day	0.92	0.66, 1.28	0.62	0.76	0.53, 1.11	0.15
03_at least 3/4 of the day	1.06	0.76, 1.47	0.73	0.66	0.46, 0.95	**0.025**
04_almost all day	0.89	0.61, 1.29	0.53	0.71	0.47, 1.07	0.10
Job Status						
01_unemployed	—	—		—	—	
02_self_employed	1.16	0.62, 2.17	0.64	1.30	0.64, 2.65	0.46
03_regularly_employed	1.22	0.67, 2.22	0.52	1.18	0.59, 2.34	0.64
wscore	0.96	0.89, 1.05	0.39	0.95	0.87, 1.04	0.25

[1]OR = Odds Ratio, CI = Confidence Interval

Appendix 2: Pain Intensity

Appendix 2.1 - Adjusted Modell - Pain Intensity

Appendix 2.1 - Adjusted Modell - Pain Intensity			
Characteristic	OR[1]	95% CI[1]	p-value
**Overall health**			
01 Very good	—	—	
02_good	1.65	1.33, 2.06	**<0.001**
03_bad	4.28	3.30, 5.58	**<0.001**
**PHQ-4**	1.27	1.22, 1.32	**<0.001**
**Activity days per week**			
00_never	—	—	
01_1day_per_wk	0.70	0.50, 0.98	**0.036**
02_2days_per_wk	0.65	0.46, 0.91	**0.011**
03_3days_per_wk	0.84	0.59, 1.20	0.34
04_four or more	0.98	0.69, 1.40	0.91
**Sport days per week**			
01 no sports	—	—	
02 less than two h per week	0.93	0.72, 1.19	0.55
03 two to 4h per week	0.89	0.69, 1.15	0.36
04 more than four h per week	0.66	0.49, 0.89	**0.007**
**Duration physical activity**			
01_less than ten minutes	—	—	
02_10 to 30 minutes	1.13	0.79, 1.62	0.50
03_30 to 60 minutes	1.06	0.74, 1.51	0.75
04_more than 60 minutes	1.24	0.86, 1.78	0.24
**Sex**			
M	—	—	
W	1.20	1.02, 1.41	**0.025**
**Age**	1.00	0.99, 1.01	0.60
**Number of M-Diagnoses**	1.06	1.04, 1.08	**<0.001**
**Number of F-Diagnoses**	0.97	0.95, 0.99	**<0.001**
**Sitting_time**			
01_less than half of the day	—	—	
02_half of the day	0.86	0.69, 1.07	0.18
03_at least 3/4 of the day	0.99	0.80, 1.23	0.92
04_almost all day	0.96	0.74, 1.23	0.72
**Job Status**			
01_unemployed	—	—	
02_self_employed	1.32	0.94, 1.85	0.11
03_regularly_employed	1.22	0.89, 1.67	0.22
**wscore**	0.98	0.93, 1.04	0.51

[1]OR = Odds Ratio, CI = Confidence Interval

Appendix 2.2 - Adjusted Modell - Pain Intensity - Sex = Female

Appendix 2.2 - Adjusted Modell - Pain Intensity - Sex = Female			
Characteristic	OR[1]	95% CI[1]	p-value
**Overall health**			
01 Very good	—	—	
02 good	1.89	1.31, 2.75	**<0.001**
03 bad	4.05	2.63, 6.28	**<0.001**
**PHQ-4**	1.25	1.18, 1.34	**<0.001**
**Activity days per week**			
00_never	—	—	
01_1day_per_wk	1.05	0.57, 1.91	0.88
02_2days_per_wk	0.76	0.40, 1.41	0.38
03_3days_per_wk	1.10	0.58, 2.10	0.77
04_four or more	1.35	0.70, 2.60	0.37
**Sport days per week**			
01_no sports	—	—	
02_less than two h per week	0.81	0.51, 1.27	0.36
03_two to 4h per week	0.71	0.44, 1.14	0.16
04 more than four h per week	0.37	0.21, 0.65	**<0.001**
**Duration physical activity**			
01_less than ten minutes	—	—	
02_10 to 30 minutes	0.98	0.51, 1.87	0.95
03_30 to 60 minutes	1.06	0.57, 1.98	0.86
04_more than 60 minutes	1.40	0.74, 2.67	0.30
**Age**	1.00	0.99, 1.01	0.84
**Number of M-Diagnoses**	1.06	1.03, 1.10	**<0.001**
**Number of F-Diagnoses**	0.97	0.94, 0.99	**0.005**
**Sitting_time**			
01_less than half of the day	—	—	
02_half of the day	0.85	0.60, 1.19	0.34
03 at least 3/4 of the day	0.90	0.62, 1.28	0.55
04 almost all day	1.01	0.66, 1.55	0.97
**Job Status**			
01_unemployed	—	—	
02_self_employed	1.18	0.74, 1.89	0.48
03_regularly_employed	1.07	0.70, 1.61	0.76
**wscore**	0.96	0.87, 1.06	0.43

[1]OR = Odds Ratio, CI = Confidence Interval

Appendix 2.3 - Adjusted Modell - Pain Intensity - Sex = Male

Appendix 2.3 - Adjusted Modell - Pain Intensity - Sex = Male			
Characteristic	OR[1]	95% CI[1]	p-value
**Overall health**			
01_Very good	—	—	
02_good	1.57	1.20, 2.08	**0.001**
03_bad	4.62	3.32, 6.46	**<0.001**
**PHQ-4**	1.28	1.22, 1.34	**<0.001**
**Activity days per week**			
00_never	—	—	
01_1day_per_wk	0.56	0.37, 0.83	**0.005**
02_2days_per_wk	0.62	0.41, 0.92	**0.017**
03_3days_per_wk	0.76	0.50, 1.15	0.19
04_four or more	0.86	0.56, 1.32	0.49
**Sport days per week**			
01_no sports	—	—	
02_less than two h per week	0.95	0.71, 1.29	0.76
03_two to 4h per week	0.97	0.71, 1.32	0.83
04_more than four h per week	0.85	0.59, 1.21	0.36
**Duration physical activity**			
01_less than ten minutes	—	—	
02_10 to 30 minutes	1.23	0.79, 1.90	0.36
03_30 to 60 minutes	1.04	0.67, 1.62	0.84
04_more than 60 minutes	1.17	0.75, 1.82	0.50
**Age**	0.99	0.98, 1.01	0.33
**Number of M-Diagnoses**	1.06	1.04, 1.08	**<0.001**
**Number of F-Diagnoses**	0.98	0.95, 1.00	**0.078**
**Sitting_time**			
01_less than half of the day	—	—	
02_half of the day	0.87	0.65, 1.16	0.33
03_at least 3/4 of the day	1.02	0.77, 1.35	0.88
04_almost all day	0.94	0.68, 1.29	0.68
**Job Status**			
01_unemployed	—	—	
02_self_employed	1.70	0.99, 2.95	**0.055**
03_regularly_employed	1.59	0.95, 2.72	**0.082**
**wscore**	0.99	0.92, 1.06	0.70

[1]OR = Odds Ratio, CI = Confidence Interval

Appendix 3: Subgroup analyses

Appendix 3.1 - Adjusted Modell - Pain Disability – Pre Covid

Appendix 3.1 - Adjusted Modell - Pain Disability – Pre Covid						
	02_moderate pain disability			03_high pain disability		
Characteristic	OR[1]	95% CI[1]	p-value	OR[1]	95% CI[1]	p-value
Overall health						
01_Very good	—	—		—	—	
02_good	1.42	0.98, 2.06	**0.064**	1.96	1.23, 3.13	**0.004**
03_bad	4.30	2.73, 6.76	**<0.001**	9.34	5.54, 15.8	**<0.001**
PHQ-4	1.16	1.08, 1.24	**<0.001**	1.42	1.33, 1.52	**<0.001**
Activity days per week						
00_never	—	—		—	—	
01_1day_per_wk	0.80	0.48, 1.34	0.41	0.68	0.40, 1.17	0.16
02_2days_per_wk	0.77	0.46, 1.29	0.32	0.80	0.47, 1.36	0.41
03_3days_per_wk	0.80	0.47, 1.37	0.42	0.85	0.48, 1.49	0.56
04_four or more	0.93	0.53, 1.62	0.79	1.23	0.69, 2.20	0.48
Sport days per week						
01_no sports	—	—		—	—	
02_less than two h per week	1.01	0.68, 1.49	0.97	0.80	0.54, 1.18	0.26
03_two to 4h per week	1.13	0.76, 1.68	0.54	0.80	0.54, 1.21	0.29
04_more than four h per week	0.71	0.45, 1.13	0.15	0.57	0.36, 0.92	**0.021**
Duration physical activity						
01_less than ten minutes	—	—		—	—	
02_10 to 30 minutes	0.76	0.43, 1.36	0.36	0.87	0.48, 1.57	0.64
03_30 to 60 minutes	0.81	0.46, 1.42	0.47	0.86	0.48, 1.55	0.62
04_more than 60 minutes	0.91	0.51, 1.61	0.74	0.80	0.44, 1.46	0.47
Sex						
M	—	—		—	—	
W	0.97	0.74, 1.26	0.80	0.95	0.72, 1.26	0.72
Age	1.00	0.99, 1.02	0.75	1.01	1.00, 1.03	0.14
Number of M-Diagnoses	1.05	1.02, 1.07	**0.002**	1.13	1.10, 1.16	**<0.001**
Number of F-Diagnoses	0.99	0.96, 1.01	0.31	0.98	0.95, 1.00	**0.090**
Sitting_time						
01_less than half of the day	—	—		—	—	
02_half of the day	0.96	0.68, 1.35	0.83	0.84	0.59, 1.20	0.34
03_at least 3/4 of the day	1.00	0.71, 1.42	0.98	0.77	0.53, 1.11	0.16
04_almost all day	0.96	0.64, 1.44	0.83	0.84	0.55, 1.29	0.44
Job Status						
01_unemployed	—	—		—	—	
02_self_employed	0.78	0.44, 1.40	0.41	0.79	0.43, 1.46	0.45
03_regularly_employed	0.94	0.54, 1.64	0.84	0.83	0.46, 1.50	0.54
Wscore	0.92	0.85, 0.99	**0.037**	0.90	0.82, 0.98	**0.011**

[1]OR = Odds Ratio, CI = Confidence Interval

Appendix 3.2 - Adjusted Modell - Pain Disability – During Covid

Appendix 3.2 - Adjusted Modell - Pain Disability - During_Covid

	02_moderate pain disability			03_high pain disability		
Characteristic	OR[1]	95% CI[1]	p-value	OR[1]	95% CI[1]	p-value
Overall health						
01_Very good	—	—		—	—	
02_good	1.98	1.43, 2.74	**<0.001**	1.42	0.88, 2.28	0.15
03_bad	4.68	2.88, 7.61	**<0.001**	7.19	3.98, 13.0	**<0.001**
PHQ-4	1.35	1.25, 1.46	**<0.001**	1.73	1.58, 1.90	**<0.001**
Activity days per week						
00_never	—	—		—	—	
01_1day_per_wk	0.49	0.25, 0.96	**0.037**	0.52	0.23, 1.19	0.12
02_2days_per_wk	0.61	0.31, 1.20	0.16	0.59	0.25, 1.37	0.22
03_3days_per_wk	0.78	0.39, 1.58	0.49	0.74	0.31, 1.81	0.51
04_four or more	0.59	0.28, 1.21	0.15	0.96	0.39, 2.36	0.94
Sport days per week						
01_no sports	—	—		—	—	
02_less than two h per week	0.73	0.44, 1.23	0.24	0.87	0.47, 1.62	0.66
03_two to 4h per week	0.62	0.36, 1.08	**0.090**	0.42	0.22, 0.83	**0.012**
04_more than four h per week	0.38	0.20, 0.71	**0.002**	0.29	0.13, 0.64	**0.002**
Duration physical activity						
01_less than ten minutes	—	—		—	—	
02_10 to 30 minutes	1.39	0.67, 2.87	0.38	1.60	0.67, 3.84	0.29
03_30 to 60 minutes	1.65	0.80, 3.40	0.18	1.43	0.60, 3.43	0.42
04_more than 60 minutes	1.78	0.86, 3.72	0.12	1.50	0.61, 3.69	0.38
Sex						
M	—	—		—	—	
W	0.86	0.66, 1.14	0.30	0.85	0.59, 1.22	0.38
Age	1.00	0.99, 1.02	0.88	1.02	1.00, 1.04	**0.057**
Number of M-Diagnoses	1.09	1.04, 1.14	**<0.001**	1.23	1.17, 1.29	**<0.001**
Number of F-Diagnoses	0.95	0.88, 1.02	0.18	0.96	0.89, 1.03	0.27
Sitting_time						
01_less than half of the day	—	—		—	—	
02_half of the day	1.10	0.73, 1.65	0.65	0.75	0.45, 1.26	0.28
03_at least 3/4 of the day	1.18	0.80, 1.74	0.41	0.63	0.38, 1.03	**0.067**
04_almost all day	0.99	0.63, 1.55	0.95	0.75	0.43, 1.33	0.33
Job Status						
01_unemployed	—	—		—	—	
02_self_employed	1.55	0.84, 2.87	0.16	1.21	0.56, 2.58	0.63
03_regularly_employed	1.18	0.68, 2.05	0.55	0.87	0.44, 1.73	0.69
wscore	1.01	0.87, 1.17	0.92	1.05	0.88, 1.26	0.61

[1]OR = Odds Ratio, CI = Confidence Interval

Appendix 3.3 - Adjusted Modell - Pain Intensity – Pre Covid

Appendix 3.3 - Adjusted Modell - Pain Intensity – Pre Covid

Characteristic	OR[1]	95% CI[1]	p-value
**Overall health**			
01_Very good	—	—	
02_good	1.32	0.94, 1.87	0.11
03_bad	3.60	2.48, 5.27	**<0.001**
**PHQ-4**	1.22	1.16, 1.28	**<0.001**
**Activity days per week**			
00_never	—	—	
01 1day per wk	0.71	0.47, 1.06	**0.10**
02 2days per wk	0.75	0.50, 1.12	0.16
03 3days per wk	0.90	0.59, 1.37	0.62
04 four or more	1.07	0.69, 1.65	0.77
**Sport days per week**			
01_no sports	—	—	
02_less than two h per week	0.89	0.66, 1.20	0.44
03_two to 4h per week	0.91	0.67, 1.24	0.55
04_more than four h per week	0.65	0.45, 0.94	**0.022**
**Duration physical activity**			
01_less than ten minutes	—	—	
02_10 to 30 minutes	1.08	0.69, 1.67	0.74
03_30 to 60 minutes	1.02	0.66, 1.57	0.94
04_more than 60 minutes	1.11	0.71, 1.72	0.66
**Sex**			
M	—	—	
W	1.14	0.92, 1.41	0.24
**Age**	1.00	0.99, 1.01	0.84
**Number of M-Diagnoses**	1.05	1.04, 1.07	**<0.001**
**Number of F-Diagnoses**	0.97	0.96, 0.99	**0.005**
**Sitting_time**			
01_less than half of the day	—	—	
02_half of the day	0.85	0.64, 1.11	0.23
03_at least 3/4 of the day	0.96	0.73, 1.27	0.79
04_almost all day	0.96	0.69, 1.34	0.82
**Job Status**			
01 unemployed	—	—	
02 self employed	1.51	0.95, 2.39	**0.079**
03 regularly employed	1.32	0.85, 2.05	0.22
**wscore**	0.97	0.91, 1.03	0.30

[1]OR = Odds Ratio, CI = Confidence Interval

Appendix 3.4 - Adjusted Modell - Pain Intensity – During Covid

Appendix 3.4 - Adjusted Modell - Pain Intensity – During Covid

Characteristic	OR[1]	95% CI[1]	p-value
**Overall health**			
01_Very good	—	—	
02_good	1.81	1.34, 2.46	**<0.001**
03_bad	4.43	2.94, 6.74	**<0.001**
**PHQ-4**	1.36	1.28, 1.46	**<0.001**
**Activity days per week**			
00_never	—	—	
01_1day_per_wk	0.72	0.39, 1.30	0.27
02_2days_per_wk	0.54	0.29, 0.98	**0.044**
03_3days_per_wk	0.79	0.42, 1.48	0.45
04_four or more	0.92	0.48, 1.76	0.81
**Sport days per week**			
01_no sports	—	—	
02_less than two h per week	1.02	0.65, 1.60	0.94
03_two to 4h per week	0.87	0.54, 1.41	0.58
04_more than four h per week	0.67	0.38, 1.17	0.16
**Duration physical activity**			
01_less than ten minutes	—	—	
02_10 to 30 minutes	1.17	0.62, 2.22	0.64
03_30 to 60 minutes	1.10	0.58, 2.09	0.76
04_more than 60 minutes	1.41	0.74, 2.71	0.30
**Sex**			
M	—	—	
W	1.31	1.02, 1.68	**0.035**
**Age**	1.00	0.99, 1.01	0.85
**Number of M-Diagnoses**	1.07	1.03, 1.11	**<0.001**
**Number of F-Diagnoses**	0.97	0.92, 1.04	0.40
**Sitting_time**			
01_less than half of the day	—	—	
02_half of the day	0.91	0.64, 1.31	0.62
03_at least 3/4 of the day	1.04	0.73, 1.48	0.82
04_almost all day	0.94	0.63, 1.41	0.78
**Job Status**			
01_unemployed	—	—	
02_self_employed	1.08	0.63, 1.86	0.79
03_regularly_employed	1.16	0.71, 1.88	0.56
**wscore**	1.03	0.90, 1.18	0.65

[1]OR = Odds Ratio, CI = Confidence Interval

Appendix 4: Sensitivity analyses

Appendix 4.1 - Adjusted Modell - Pain Disability – Pre Covid - Sensitivity

Appendix 4.1 - Adjusted Modell - Pain Disability – Pre Covid - Sensitivity

	02_moderate pain disability			03_high pain disability		
Characteristic	OR[1]	95% CI[1]	p-value	OR[1]	95% CI[1]	p-value
Overall health						
01_Very good	—	—		—	—	
02_good	1.55	0.98, 2.46	**0.062**	2.80	1.52, 5.15	**<0.001**
03_bad	4.31	2.48, 7.50	**<0.001**	13.1	6.71, 25.7	**<0.001**
PHQ-4	1.12	1.03, 1.22	**0.006**	1.44	1.33, 1.56	**<0.001**
Activity days per week						
00_never	—	—		—	—	
01_1day_per_wk	0.77	0.41, 1.47	0.43	0.73	0.37, 1.44	0.36
02_2days_per_wk	0.65	0.34, 1.24	0.19	0.72	0.37, 1.40	0.33
03_3days_per_wk	0.59	0.30, 1.14	0.12	0.71	0.35, 1.43	0.34
04_four or more	0.79	0.39, 1.57	0.49	1.03	0.51, 2.12	0.93
Sport days per week						
01_no sports	—	—		—	—	
02_less than two h per week	0.97	0.61, 1.56	0.91	0.78	0.48, 1.25	0.30
03_two to 4h per week	1.27	0.79, 2.06	0.33	0.98	0.60, 1.59	0.92
04_more than four h per week	0.77	0.44, 1.35	0.36	0.66	0.37, 1.18	0.17
Duration physical activity						
01_less than ten minutes	—	—		—	—	
02_10 to 30 minutes	0.89	0.44, 1.80	0.75	1.03	0.50, 2.12	0.94
03_30 to 60 minutes	1.06	0.53, 2.12	0.87	1.06	0.52, 2.16	0.88
04_more than 60 minutes	1.06	0.53, 2.14	0.86	1.14	0.55, 2.34	0.73
Sex						
M	—	—		—	—	
W	0.90	0.65, 1.26	0.54	1.02	0.72, 1.44	0.93
Age	1.00	0.98, 1.02	0.88	1.01	0.99, 1.03	0.53
Number of M-Diagnoses	1.05	1.01, 1.08	**0.013**	1.16	1.12, 1.20	**<0.001**
Number of F-Diagnoses	0.99	0.97, 1.02	0.66	0.97	0.95, 1.00	**0.055**
Sitting_time						
01_less than half of the day	—	—		—	—	
02_half of the day	0.93	0.62, 1.39	0.71	0.71	0.46, 1.10	0.12
03_at least 3/4 of the day	0.93	0.61, 1.41	0.72	0.85	0.55, 1.32	0.47
04_almost all day	0.71	0.42, 1.17	0.18	0.72	0.43, 1.22	0.23
Job Status						
01_unemployed	—	—		—	—	
02_self_employed	0.80	0.40, 1.62	0.54	0.69	0.34, 1.42	0.31
03_regularly_employed	0.89	0.46, 1.74	0.74	0.73	0.37, 1.45	0.37
wscore	0.94	0.85, 1.04	0.21	0.92	0.83, 1.02	0.10

[1]OR = Odds Ratio, CI = Confidence Interval

Appendix 4.2 - Adjusted Modell - Pain Disability – During Covid Sensitivity

Appendix 4.2 - Adjusted Modell - Pain Disability – During Covid-Sensitivity

	02_moderate pain disability			03_high pain disability		
Characteristic	OR[1]	95% CI[1]	p-value	OR[1]	95% CI[1]	p-value
Overall health						
01_Very good	—	—		—	—	
02_good	2.00	1.43, 2.79	**<0.001**	1.45	0.88, 2.39	0.15
03_bad	4.95	2.97, 8.26	**<0.001**	7.60	4.07, 14.2	**<0.001**
PHQ-4	1.33	1.22, 1.44	**<0.001**	1.67	1.52, 1.84	**<0.001**
Activity days per week						
00_never	—	—		—	—	
01_1day_per_wk	0.50	0.25, 0.99	**0.047**	0.53	0.22, 1.28	0.16
02_2days_per_wk	0.59	0.30, 1.19	0.14	0.59	0.24, 1.45	0.25
03_3days_per_wk	0.75	0.36, 1.55	0.43	0.87	0.34, 2.22	0.76
04_four or more	0.61	0.29, 1.29	0.19	0.99	0.38, 2.57	0.99
Sport days per week						
01_no sports	—	—		—	—	
02_less than two h per week	0.77	0.45, 1.32	0.34	0.86	0.44, 1.67	0.65
03_two to 4h per week	0.64	0.36, 1.13	0.12	0.42	0.20, 0.86	**0.018**
04_more than four h per week	0.39	0.20, 0.76	**0.006**	0.30	0.13, 0.68	**0.004**
Duration physical activity						
01_less than ten minutes	—	—		—	—	
02_10 to 30 minutes	1.12	0.52, 2.37	0.78	1.14	0.45, 2.86	0.78
03_30 to 60 minutes	1.44	0.68, 3.04	0.34	1.05	0.41, 2.63	0.92
04_more than 60 minutes	1.52	0.71, 3.25	0.28	1.12	0.43, 2.89	0.82
Sex						
M	—	—		—	—	
W	0.86	0.64, 1.14	0.28	0.90	0.61, 1.32	0.58
Age	1.00	0.99, 1.02	0.81	1.02	1.00, 1.04	0.14
Number of M-Diagnoses	1.09	1.04, 1.14	**<0.001**	1.23	1.17, 1.30	**<0.001**
Number of F-Diagnoses	0.95	0.87, 1.04	0.26	0.98	0.89, 1.07	0.60
Sitting_time						
01_less than half of the day	—	—		—	—	
02_half of the day	1.09	0.71, 1.65	0.70	0.75	0.44, 1.30	0.31
03_at least 3/4 of the day	1.16	0.77, 1.74	0.47	0.63	0.37, 1.07	**0.086**
04_almost all day	0.99	0.62, 1.58	0.96	0.74	0.40, 1.35	0.32
Job Status						
01_unemployed	—	—		—	—	
02_self_employed	1.42	0.74, 2.72	0.29	1.00	0.44, 2.29	>0.99
03_regularly_employed	1.08	0.60, 1.92	0.81	0.79	0.38, 1.66	0.54
wscore	1.04	0.88, 1.22	0.66	0.99	0.79, 1.23	0.91

[1]OR = Odds Ratio, CI = Confidence Interval

Appendix 4.3 - Adjusted Modell - Pain Intensity – Pre Covid - Sensitivity

Appendix 4.3 - Adjusted Modell - Pain Intensity – Pre Covid - Sensitivity

Characteristic	OR[1]	95% CI[1]	p-value
**Overall health**			
01_Very good	—	—	
02_good	1.55	1.00, 2.43	**0.052**
03_bad	4.46	2.78, 7.28	**<0.001**
**PHQ-4**	1.24	1.18, 1.32	**<0.001**
**Activity days per week**			
00_never	—	—	
01_1day_per_wk	0.74	0.44, 1.23	0.25
02_2days_per_wk	0.68	0.41, 1.13	0.13
03_3days_per_wk	0.74	0.43, 1.27	0.27
04_four or more	0.97	0.56, 1.67	0.92
**Sport days per week**			
01_no sports	—	—	
02_less than two h per week	0.86	0.60, 1.24	0.43
03_two to 4h per week	1.07	0.74, 1.55	0.73
04_more than four h per week	0.69	0.44, 1.07	0.10
**Duration physical activity**			
01_less than ten minutes	—	—	
02_10 to 30 minutes	1.18	0.68, 2.04	0.55
03_30 to 60 minutes	1.07	0.62, 1.84	0.80
04_more than 60 minutes	1.25	0.72, 2.16	0.42
**Sex**			
M	—	—	
W	1.03	0.79, 1.35	0.83
**Age**	1.01	0.99, 1.02	0.36
**Number of M-Diagnoses**	1.06	1.04, 1.09	**<0.001**
**Number of F-Diagnoses**	0.98	0.96, 1.00	**0.030**
**Sitting_time**			
01_less than half of the day	—	—	
02_half of the day	0.77	0.55, 1.07	0.12
03_at least 3/4 of the day	1.00	0.71, 1.40	0.98
04_almost all day	0.92	0.61, 1.38	0.68
**Job Status**			
01_unemployed	—	—	
02_self_employed	1.51	0.87, 2.63	0.15
03_regularly_employed	1.41	0.83, 2.40	0.20
**wscore**	0.97	0.90, 1.05	0.50

[1]OR = Odds Ratio, CI = Confidence Interval

Appendix 4.4 - Adjusted Modell - Pain Intensity – During Covid - Sensitivity

Appendix 4.4 - Adjusted Modell - Pain Intensity – During Covid - Sensitivity

Characteristic	OR[1]	95% CI[1]	p-value
**Overall health**			
01_Very good	—	—	
02_good	1.84	1.35, 2.53	**<0.001**
03_bad	4.52	2.93, 7.05	**<0.001**
**PHQ-4**	1.36	1.27, 1.46	**<0.001**
**Activity days per week**			
00_never	—	—	
01_1day_per_wk	0.71	0.38, 1.34	0.29
02_2days_per_wk	0.47	0.25, 0.89	**0.020**
03_3days_per_wk	0.68	0.35, 1.32	0.26
04_four or more	0.78	0.40, 1.54	0.48
**Sport days per week**			
01_no sports	—	—	
02_less than two h per week	1.03	0.63, 1.67	0.90
03_two to 4h per week	0.93	0.55, 1.56	0.78
04_more than four h per week	0.77	0.42, 1.40	0.39
**Duration physical activity**			
01_less than ten minutes	—	—	
02_10 to 30 minutes	1.01	0.52, 1.99	0.97
03_30 to 60 minutes	1.02	0.52, 2.00	0.95
04_more than 60 minutes	1.27	0.64, 2.51	0.49
**Sex**			
M	—	—	
W	1.30	1.00, 1.69	**0.049**
**Age**	1.00	0.98, 1.01	0.60
**Number of M-Diagnoses**	1.07	1.03, 1.12	**<0.001**
**Number of F-Diagnoses**	0.97	0.91, 1.04	0.39
**Sitting_time**			
01_less than half of the day	—	—	
02_half of the day	0.88	0.60, 1.28	0.50
03_at least 3/4 of the day	0.99	0.69, 1.43	0.95
04_almost all day	0.91	0.59, 1.39	0.66
**Job Status**			
01_unemployed	—	—	
02_self_employed	1.14	0.63, 2.05	0.67
03_regularly_employed	1.18	0.70, 2.01	0.53
**wscore**	1.02	0.88, 1.19	0.77

[1]OR = Odds Ratio, CI = Confidence Interval
